# Effect of Cereclor as Rejuvenator to Enhance the Aging Resistance of Reclaimed Asphalt Pavement Binder

**DOI:** 10.3390/ma13071582

**Published:** 2020-03-30

**Authors:** Ghulam Yaseen, Imran Hafeez

**Affiliations:** 1 Department of Civil Engineering, The University of Lahore (Islamabad Campus), Islamabad 44000, Pakistan; 2 Department of Civil Engineering, University of Engineering and Technology Taxila, Rawalpindi 46000, Pakistan; imran.hafeez@uettaxila.edu.pk

**Keywords:** cereclor, rejuvenator, colloidal system, physical-rheological properties, aging resistance, pavement recycling

## Abstract

Asphalt is the most commonly used material for pavement construction around the world, and therefore, it is vital to acquaint a practice that restores the reclaimed asphalt pavement (RAP) binder properties to the required level of performance by adding proper rejuvenators. However, a rejuvenator may perform better in the early stages of its application but may not necessarily perform better in the long run. The aim of this study is to assess the rejuvenation effect on the aging resistance of RAP binder in long-life performance through applying artificial aging. In this study, base virgin binder of pen grade 60/70 and RAP binder rejuvenated with Cereclor were subjected to artificial aging to simulate the short- and long-term aging effects. Penetration, softening point, ductility, and viscosity; saturates, aromatics, resins, and asphaltene (SARA) fractionation; and Fourier-transform infrared (FTIR) spectroscopy, bending beam rheometer (BBR), and dynamic shear rheometer (DSR) tests were conducted to evaluate the potential improvements in various properties of RAP binder. The results indicated that the physical, fractional composition, rheological, and aging resistance of RAP binder improved through the rejuvenation mechanism. Therefore, the rejuvenator improved the chemical structure through re-balancing the constituents in the colloidal system, even after long-term re-aging, which proves it to be an aging-resistant binder. Furthermore, it has been concluded that Cereclor has substantial rejuvenation potential even after applying artificial aging, and it can be utilized in pavement recycling to achieve long-life performance. Furthermore, the results depict good correlations between the physical, rheological, and chemical parameters of virgin and RAP binder.

## 1. Introduction

Declining natural resources have urged for the sustainable construction and recycling of constituent pavement materials. Up until now, asphalt pavements have been attaining the milestone of new roads and the rehabilitation of existing roads in Pakistan. The material extracted from the removal, milling action, and crushing of existing pavements is termed as reclaimed asphalt pavement (RAP) and is mostly recycled. Recycling of asphalt pavement is a valuable technique to target economic and sustainable development in pavement engineering [1]. Utilization of RAP initially started in the early 1980s in the United States of America (USA) with the analysis of several factors and then spread globally with energy constraints [2,3]. Prior literature offers a significant indication of recycled materials used in pavement construction in Illinois [4,5,6].

The application of RAP provides several economic paybacks to replace the virgin asphalt binder. Still, as its quantity increases, the manufacture and in-service performance of the remolded pavements have become great challenges because of the aged and brittle binder. To reduce the hardness of asphalt, rejuvenators are incorporated into the asphalt mixtures for optimum results [2,4]. The recycled binders in RAP are comparatively more aged and stiffer, which may cause early accelerating distresses but enhance the rutting resistance [7,8,9].

The failure of asphalt pavements due to aging is a hot topic in pavement construction and technology. The excellence and long life of the pavements are dependent on the extent of aging due to the asphalt binder in the final hot mixture asphalt [10]. There are two main theories such as compositional harmonic and compatibility theory for the regeneration mechanism of rejuvenators. The compositional harmonic theory describes that asphalt binder exists as a colloidal solution in which each constituent is distributed in a fixed location in an unaged condition [11,12,13]. During aging, constituents’ relative content changes and governs the degradation of different properties in asphalt binder that governs performance life. The content of different constituents in the aged binder is restored through the addition of various types of rejuvenators [14].

According to compatibility theory, asphalt binder is like a dispersion system in which asphaltene is the dispersing agent and maltene is the dispersoid. Both contents are uniform in the unaged binder, thereby establishing a stable dispersion mechanism [15]. Asphaltene content is increased due to aging, in which the entire phenomenon of aging comprises intrinsic variables such as binder and aggregate properties, binder content in the hot mix asphalt (HMA) mixture, air voids, and binder coating layer thickness; the variables of extrinsic nature include mixing temperature, effects of the environment (ultraviolet radiation, temperature variation, intensity of rainfall), and exposure time that simulates the aging conditions occurring during the service life of pavements [12,16]. Different rejuvenation mechanisms exist that contribute to the healing of the performance properties of the aged binder.

Reversible fluxing in bitumen, RAP binder, and warm mix asphalt is possible by adding different types of rejuvenators. The systematic investigation of the mechanical, rheological, aging, and temperature sensitivity, as well as the morphology and thermal properties of various polymer-modified binders (PMBs), have a few advantages and drawbacks [17]. It has been shown that polymer modification enhances some characteristics of asphalt binder such as better elastic recovery, cracking resistance at low temperature, and high rutting resistance at elevated temperature. Meanwhile, these polymers have exhibited thermal instability and phase segregation issues [18,19]. Furthermore, [20,21] showed that vegetable oil produced from raw materials (rapeseed oil, soybean oil, sunflower oil, linseed oil, canola oil, coconut oil, castor oil, etc.) and mixed in asphalt binder acts as modifiers/additives with different percentages [22]. It is concluded that waste cooking oil (WCO) as rejuvenators is useful for their low cost and fewer environmental concerns. From a chemical point of view, oil exposed to higher temperature alters its chemical composition and produces high anti-oxidant molecules used as rejuvenators in asphalt binder [23]. However, WCO has certain drawbacks, such as weak low-temperature fluid characteristics, the tendency of oxidative degradation, and vulnerability to hydrolysis in acidic media, which bounds its applications. A unique rejuvenator “Cereclor” with different percentages was utilized to enhance the performance properties of RAP binder, as well as to replace the traditional emulsifiers. After addition, it rejuvenates the RAP binder and improves the fatigue and aging resistance of bituminous mixtures [24].

To date, various chemical additives have been utilized for the target of the binder modification of organo-metallic compounds, sulfur, polyphosphoric acid, sulfonic acid, and reactive polymers. However, few of the above-mentioned chemical compounds have been utilized practically [25]. Sulfur is available in several allotropic shapes such as monoclinic, rhombic, and polymeric, which diverge in physical and chemical characteristics. As sulfur is added with asphalt binder, it can react in different patterns depending upon the quantity of sulfur and the mixing temperature. The chemical reaction between sulfur and asphalt binder is like a vulcanization process at high temperatures. However, generally lower than 140 °C, it could be combined, chemically making polysulfides, in which non-reacted sulfur dissolves [26]. In the literature, [27,28] describes that generally 20% sulfur can be dissolved in asphalt binder and a higher quantity of sulfur exists in crystalline condition.

The sole purpose of asphalt binder modification is to control the lower-service-temperature areas that desire less stiffness and rapid relaxation characteristics to minimize the cracks that occur at low temperature. Asphalt binders perform effectively under specific conditions such as lower viscosity to facilitate pumping actions and mixing and compaction of HMA at high construction temperatures. However, during the service life of pavements, a greater stiffness is required to resist shoving and rutting. Most of these norms could be achieved through modifiers [29]. Such motives encouraged the pavement specialists to introduce new rejuvenators to improve the performance properties of RAP binders at a wide range of temperatures [30].

In summary, it is the consensus that the incorporation of different types of rejuvenators might provide a major role to promote the utilization of RAP materials in pavement recycling. However, several rejuvenators were utilized in previous studies to restore the colloidal structure of the aged binder that could contribute to the enhancement of the performance properties. Moreover, a rejuvenator may perform better at an early stage of its application but may not necessarily perform better in the long run. Due to such reasons, the exploration of new rejuvenators that restore the colloidal structure of aged binders, as well as efficiently perform in the long run in developing countries such as Pakistan, is still absent. Hence, there is a need to explore the effects of a new rejuvenator along with a chemical agent that effectively contributes to the long-life performance of pavement recycling.

Thus, a new concept was introduced to use Cereclor (C) as a rejuvenator to enhance a saturated portion of the maltene phase that should balance the dispersion of asphaltene and maltene phases to ensure optimum results at low, intermediate, and high temperatures. According to different theories of governing rejuvenating mechanisms, it can be hypothesized that if RAP binder (RB) is rejuvenated to improve the aliphatic chains containing hydrocarbons, it might lower the viscosity and stiffness due to the higher fraction of the maltene phase. Additionally, due to the lack of unsaturated hydrocarbons in the colloidal structure of asphalt binder, it contributes to a higher aging resistance. The new rejuvenator ‘Cereclor’ with eminent features was selected depending upon this idea as compared to other rejuvenators.

Chlorinated paraffin (CP) or polychlorinated n-alkanes (PCAs) commonly known as “Cereclor” have the general formula *(CnH2n + 2 − zClz)* and are composite blends comprising thousands of various isomers, diastereomers, and enantiomers. CPs are further subcategorized according to their carbon chain length from short to medium to long chains. The physical and chemical characteristics of these mixtures are associated with carbon chain linkage and percent chlorination from 10% to 72%. It can be prepared through the chlorination process in a batch of liquid n-alkanes with high purity through an exothermic reaction in the presence of hydrogen chloride [31].

The carbon chain length and percentage of chlorine content play a major rule in the physical and chemical properties of Cereclor. These materials are complex mixtures of homologs and isomers that exist in the liquid phase with a light-yellow color and are insoluble in water but can form an emulsion or suspension. The melting point of Cereclor increases with carbon chain length and chlorine content [32]. It can be added to various categories of sealants and paints to increase their life due to resistance against the attack of water. It can also be used in marine paints and industrial flooring, vessels, swimming pools, and paints for pavement surface marking.

In addition to the physical blending of RB and rejuvenators, another way to enhance this blend of characteristics is through chemical modification, which utilizes the chemical additives to modify the performance properties through cross-linking. In such a scenario, this study would explore the effect through the addition of “Cereclor” as a rejuvenator and “sulfur” as a chemical agent before and after applying the aging process by performing physical, rheological, and physicochemical test methods. The key objective of this study is to explore and contribute to the hybrid rejuvenator mechanism through improving the performance of rejuvenated aged binders that might contribute to the pavement recycling industry. Finally, there is limited to no research carried out to evaluate the different types of correlations between the physical, rheological, and chemical parameters of asphalt binder. One of the aims of this research study is to establish such correlations between physical, rheological, and chemical parameters in the presence of the new rejuvenator to enhance the performance of asphaltic pavements.

## 2. Objectives of the Research

The primary aims and purposes of this study were to characterize the contribution of the new rejuvenator on RAP binder (RB) properties after applying further artificial aging. However, these key points are mentioned herein:

- To characterize the colloidal instability of Virgin Binder pen grade 60/70 (VB), RAP binder, and Rejuvenated RAP binder after applying the artificial aging through fractional composition.
- To depict the effect of rejuvenation on the aging resistance of Virgin, RAP binder, and Rejuvenated RAP binder after applying the artificial aging.
- To investigate the effect of rejuvenation on rheological characteristics of Virgin, RAP binder and Rejuvenated RAP binder after applying the artificial aging.
- To establish correlations between the physical, rheological, and chemical parameters of Virgin, RAP binder, and Rejuvenated RAP binder after applying the artificial aging.

## 3. Materials and Methods

The entire methodology is described by three phases such as collection of materials, rejuvenation, and testing program. In the 1st phase, virgin binder (VB) penetration grade 60/70 was collected from Attock Refinery Limited (ARL), and standard artificial aging tests were applied to simulate the short- and long-term aging. Samples were prepared and conditioned for the rolling thin film oven test (RTFOT), designated as ‘VBSTA.’ After the RTFOT, samples were conditioned with a pressure aging vessel (PAV) at 2.10 MPa of pressure and 100 °C for 20 h to simulate the long-term aging in the service life of pavements, designated as ‘VBLTA.’

**Figure 1:**
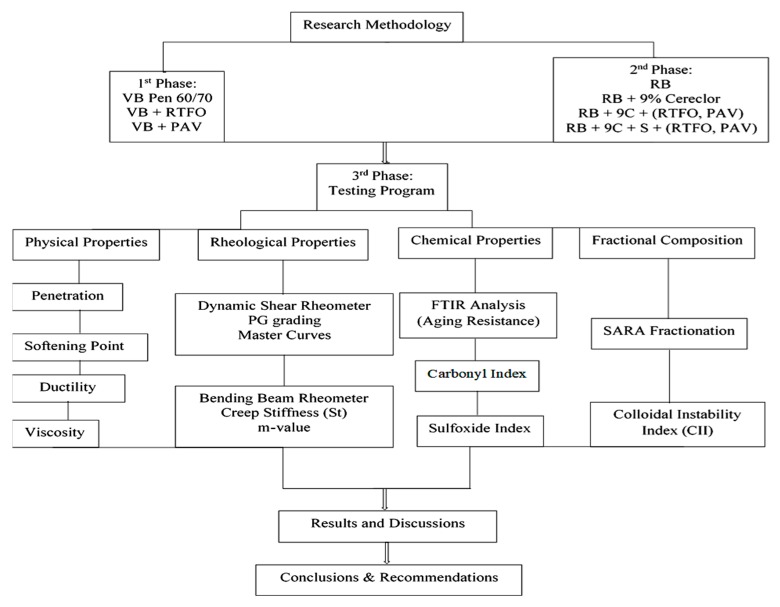
Schematic diagram of the entire methodology.

In the 2nd phase, RB was extracted from RAP Material taken from Motorway M-2 using a centrifuge and Rota vapor equipment according to ASTM D2172 and ASTM D5404, respectively. RB was rejuvenated through the optimum dose of Cereclor. The inclusive mechanism of the rejuvenation of RB along with the addition of sulfur was performed as discussed in the next section. The rejuvenated blends (RB, RB9C, RB9C0.5S, RB9C1S, and RB9C1.5S) were also subjected to artificial aging through RTFO and PAV to measure and compare its properties to the parent binder. In the 3rd phase, testing was conducted to evaluate basic physical properties, SARA fractions, flow characterization, aging resistance, and rheological properties, at low, intermediate, and high temperatures on virgin and rejuvenated RB along with the addition of sulfur. The entire methodology of this research study is described in Figure 1.

### 3.1. Materials

Pakistan is geographically located in an area where weather conditions hit extreme limits in the summer and winter seasons. Asphalt binders of different penetration grades are produced by two local oil refineries, Attock Refinery Limited (ARL-North region) and National Refinery Limited (NRL-south region). Penetration grade 60/70 of ARL was utilized in the construction of the Islamabad-Lahore Motorway (M-2) of about 376 km in 1996. It was initially designed for 30 million equivalent single-axle loads. To achieve suitable serviceability, routine maintenance continued periodically. After completion of 20 years’ service life, rehabilitation was recommended through non-destructive testing. During the rehabilitation, more than one-million tons of RAP binder (RB) was produced. In this research study, the following materials such as virgin binder of penetration grade 60/70 from ARL and RAP material from M-2, Cereclor as rejuvenator and Sulfur as cross-linking agent in the rejuvenated RB were utilized. Physical properties of all these materials is shown in Table 1.

### 3.2. Rejuvenation

Various types of rejuvenators have been utilized that can replenish the ingredients lost during the aging and have supposed different interpretations of the regeneration mechanism of asphalt binder. In a previous study, the optimum dose of Cereclor was determined for recycling [24]. Cereclor was chosen, different trials were applied, and 9% (optimum dosage) was selected to explore its effects on RB properties after applying the rolling thin film oven (RTFO) and pressure aging vessel (PAV) aging processes and with reference binder. To ensure the compatibility, it can be noted that Cereclor is also having similar in nature and chemical function like asphalt binder. The major composition of both materials is hydrocarbons and also contains polar and non-polar constituents. The polarity in asphalt binder is governed through oxygen, including sulfur along with a few metals, while chlorine atoms are attached with carbons atoms that induce polarity in Cereclor with the remaining C–H bonds having a non-polar nature. Due to this reason, as Cereclor is mixed with asphalt binder, polar and non-polar bonds are attracted to dissolve each other. This concept was introduced to rejuvenate the RAP binder for inducing polarity through a chlorine atom, which ultimately balances the asphaltene and maltene fractions to enhance performance properties. Cereclor was added in the RB at 140 °C at a rate of 3000 rpm with continuous stirring for 15 min at controlled conditions to make the homogeneous blend and was abbreviated as “RB9C.”

In addition to a physical blending of RB and Cereclor, different dosages of sulfur (0.5%, 1%, 1.5%) were also added in that blend at 140 °C and mechanically continuously stirred at 3000 rpm for 30 min at controlled conditions for suitable mixing. The rule of sulfur in the chemical structure of asphalt binder and its properties are quite versatile and have not been studied completely to date. After mixing, the reaction depends on the temperature range and content of sulfur. It looks like an octet ring at the melting point and forms into the 2-radical chain at higher temperatures. These radicals can react with asphalt binder in two different scenarios: Establishing a carbon–sulfur bond or absorbing hydrogen and finally undergoing dehydrogenation. The probability of such reactions relies on temperature; therefore, the product might change. At higher temperatures, it acts as oxygen and the reaction leads to oxidation, releasing hydrogen disulfide. Dehydrogenation chains are converted by cyclization, finally increasing the asphaltene content and acting like the vulcanization process in the presence of polymers. Meanwhile, at 140 °C, as the reaction takes place, sulfur atoms are entered into aromatic naphthalene molecules and reduce their quantity in the asphalt binder. During this phenomenon, polar aromatic groups are increased to enhance their adhesive and cross-linking properties. At this stage, sulfur-binder solvation, suspension of its excess quantity as colloidal particles, and its tendency to precipitate also exist, which makes it more useful. In this research study, physical blending was carried out at 140 °C to enhance the cross-linking properties in the rejuvenated blend of the RB.

### 3.3. Testing Methods

Initially, standard laboratory aging tests were utilized to simulate the short- and long-term aging on virgin binder pen 60/70 samples. Samples were prepared, conditioned, and placed in rolling glass jars of the rolling thin film oven test (RTFOT) ASTM D2872 apparatus at 163 °C for 85 min by undergoing oxidation with the airflow of 400 mL/min. After the RFTOT, samples were conditioned with a pressure aging vessel (PAV) with a pressure of 2.10 MPa and temperature of 100 °C for 20 h to simulate the long-term aging in the service life of pavements. Conventional physical properties of asphalt binder such as penetration, softening point, ductility, loss on heating, and specific gravity were determined according to AASHTO T 29, AASHTO T 53, AASHTO T 51, and AASHTO T 228, respectively. Asphalt binder is a viscoelastic-plastic material and its resistance to flow under temperature variation is called viscosity. The Brookfield rotational viscometer test (ASTM D 4402-02) was conducted to measure the viscosity of asphalt binder at 135 °C.

Separation of a general fraction of asphalt binder into four main fractions was carried out according to ASTM D4124-09. Nearly 10 g (± 1) from each Virgin, RB and Rejuvenated RB before and after applying the artificial aging were taken one by one in the flask in the presence of n-heptane at a ratio of 30 mL per gram of binder. The flask was placed onto a heating plate and ignited with stirring to 90 °C. The blend was shaken until no binder was stuck inside the flask. Later, heating and stirring continued for about 1.5 h. After that, the flask was detached from the plate, locked, and placed in the dark overnight. The next day, n-heptane was filtered through a suitable funnel. The liquid phase was placed in an additional flask, properly wrapped to evade oxidation. The remaining solidified material was washed twice with 100 mL n-heptane at normal lab temperature. Furthermore, this solid material or constituent was sealed in a filter and placed in a Soxhlet extractor in a solvent of 750 mL n-heptane. To determine the properly purified asphaltene, the extraction was prolonged for up to 48 h and the reflow was checked by dropping a single drop of the liquid onto the glassy plate.

During this, if there was no visible stain on the plate with the later evaporation of the solvent, the extraction process was ended; otherwise, it could be prolonged for the next 24 h. The two n-heptane unsolvable phases were combined and enclosed in filter paper and responded to toluene extraction with 750 mL toluene in a similar extractor. The toluene was evaporated, and the residue material was examined. Time slot and abortion conditions were identical for the n-heptane extraction. Similarly, the liquid phase was filtered, and double n-heptane phases were combined and concentrated to obtain 50 mL (± 5mL). It was moved onto a chromatographic column occupied with 450 g of dry aluminum oxide up to 80 cm (±1 cm) length and prewetted with 400 mL of n-heptane. The entire procedure was selected for each sample according to ASTM D4124-09. However, because of the prewetting of the column, forerunnings of 350 mL were gathered.

A FTIR (6700 Nicolet) spectrometer was used to measure the binder spectra in the wavenumber range of 400–4000 cm^−1^. For spectroscopic examination, a small quantity of each asphalt binder sample (10 mg) was placed directly on the diamond crystal with a clean metal spatula. The spectra were collected according to the specified range with a resolution of 4 cm^−1^, and every spectrum denoted an accumulation of 32 spectra. For detecting the aging process for the aged binder, it accurately showed the alteration in intensity of the spectral peaks, confirming carbonyl peaks around 1700 cm^−1^ and a sulfoxide functional group at 1030 cm^−1^.

The bending beam rheometer (BBR) test was performed on selected samples according to AASHTO M320, and the flexural creep stiffness was evaluated to measure the low-temperature cracking critical temperature. The two basic parameters were measured on samples through engineering beam theory at a fixed creep load. These parameters are the creep stiffness (S) and creep rate or the rate of stress relaxation, usually termed as the m-value or slope of the creep stiffness master curve asphalt binders that are not too stiff at low temperature and that govern the relaxation build-up of stresses to resist excessive thermal cracking. It is elaborated at a loading time of 60 s. The creep stiffness should not be higher than 300 MPa, and the m-value should be at least 0.3 to meet the requirements for low-temperature cracking resistance.

Performance Grading (PG) grading was performed on a dynamic shear rheometer (DSR) to determine the upper-temperature grade of different samples of asphalt binder in accordance with AASHTO M320. It measured the high-temperature grade in strain-controlled mode (10% strain) at a frequency level of 10 rad/s. The specimen response to sinusoidal stress was measured in the form of a complex shear modulus (G*) and a phase angle (δ). Frequency sweep tests were conducted on different aged samples to measure the complex shear modulus (G*) and phase angle (δ) at intermediate and high temperatures (22 °C, 34 °C, 46 °C, 58 °C, 70 °C, 82 °C) at a frequency range between 0.1 and 10 rad/s using the Anton Paar Peltier-type dynamic shear rheometer (DSR) to plot the master curves of the prepared samples.

## 4. Results and Discussion

### 4.1. Conventional Properties of the Virgin, Aged, and Rejuvenated Aged Binders

Conventional physical properties of different asphalt binders such as penetration, softening point, ductility, loss on heating, and specific gravity were determined, and the results are tabulated in Table 2.

It can be observed from Table 2 that penetration and ductility decreased, and the softening point increased significantly from VB to VBSTA, VBLTA, and RB. With the addition of Cereclor and sulfur and applying both aging processes, penetration was still in reference-grade with reasonable ductility and minor changes in the softening point. It can be noted that the rejuvenators significantly softened it and might have restored the distribution of constituent in a colloidal solution like an unaged binder. This rejuvenation mechanism may also induce polarity through a chlorine atom, which would ultimately balance the asphaltene and maltene fractions. With higher fractions of oily maltenes, the molecules are free to move in the system of fluid that improves its temperature susceptibility, as referred to in the literature [33].

**Figure 2:**
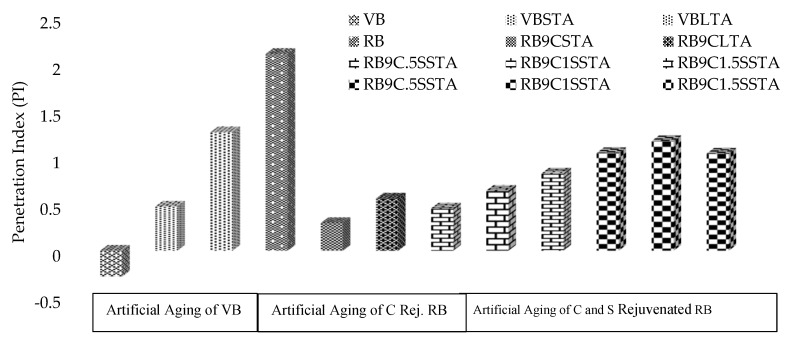
Effect of rejuvenators and aging process on penetration index (PI) of asphalt binders.

Figure 2 reveals the penetration index (PI) of different samples, as described in the 1st and 2nd phase of the methodology. The general value of the PI ranges from −3 for high temperature-susceptible to +7 for highly blown low temperature-susceptible (high PI) asphalt binders, as described in the literature [34].

It can be noted from Figure 2 that the PI values increased with aging and RB contained the highest value, i.e., 2.11, indicating less low-temperature susceptibility. Through the addition of rejuvenator and applying the artificial aging processes, it lowers the PI value significantly from 2.11 to 0.47 and is still less than the PI value of VBLTA. This trend may also indicate less low-temperature susceptibility. The addition of different dosages of sulfur, after aging the samples, depicts minor increases in PI on all samples, but still less than the VBLTA and RB values. Furthermore, the ‘PI’ parameter uses only two temperature references, so for better simulation of results, viscosity and stiffness tests may be utilized. This criterion is normally recommended in the construction of asphaltic pavements as it replicates the better resistance to loading and deformation, as mentioned in the literature [35].

### 4.2. Chemical Composition of Virgin, Aged, and Rejuvenated Aged Binders by SARA Fractionation

**Figure 3:**
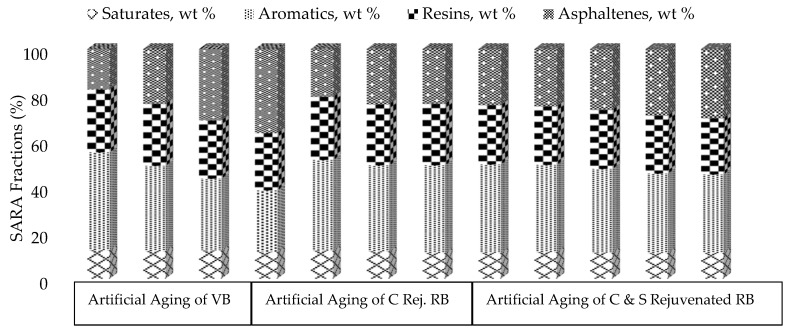
Effect of rejuvenation and aging on saturates, aromatics, resins, and asphaltene (SARA) fractions of binder.

The separation of a general fraction of asphalt binder into four main fractions was carried out, and the results are presented in Figure 3.

It may be noted from Figure 3 that in the 1st phase, the saturated contents slightly changed but the aromatics decreased, which significantly increased the asphaltene and resins content in VBSTA and VBLTA samples. A similar trend is also observed in RB, which contains the highest percentage of asphaltene content due to the adverse aging in the field during the last ten years. During the aging, the entire colloidal solution and distribution of constituents altered, ultimately hardening and stiffening the binder. Through the addition of rejuvenator in the RB, it can be noted that the distribution of fractions such as asphaltene content decreased and the aromatic content increased significantly. This may be happened due to change in the dispersion system of asphaltene and maltene, as well as varying the constituents in colloidal solution in RB. The effect of the aging process along with rejuvenation was also considered, and still, it can be observed that the asphaltene content was less than in the VBLTA sample. Furthermore, through the addition of sulfur in the rejuvenated RB and applying the aging process, the asphaltene and aromatic content changed. Still, these fractions are lower than those of the VBLTA and RB samples. It can be depicted that this novel approach has significantly changed the distribution of fractions and resistance to aging. Meanwhile, such changes in SARA fractions can be elaborated through the oxidation process that happens during aging, as evidenced by infrared spectra.

From the SARA fractions, the colloidal instability index (CII) was calculated. This index represented as the ratio of dispersed fractions (aromatics and resins) to flocculated fractions (saturates and asphaltenes) is a gauge to detect the colloidal equilibrium in the form of a sol or gel structure in the asphalt binders, as given in Equation (1).

(1) $$Collidal\;Instability\;Index\;\left(CII\right)=\frac{Floculated}{Dispersed}=\frac{Saturates+Asphaltenes}{Resins+Aromatics}$$

**Figure 4:**
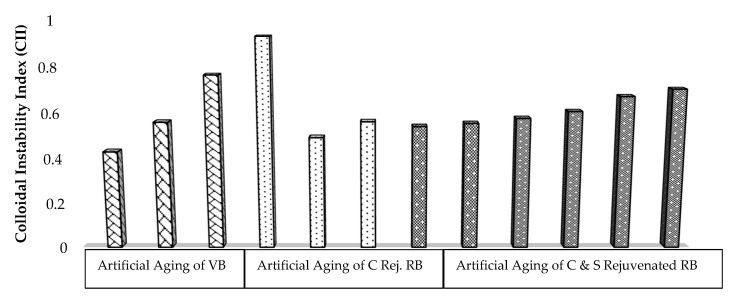
Effect of rejuvenation and aging on colloidal instability index (CII) of binders.

Its higher values indicate the stiffer binder as the asphaltenes are more peptized through the resins, which govern the sol structure, while a lower value softens the binder that describes the gel structure [36]. Furthermore, the variation in fractional contents through SARA analysis and CII for different samples is represented graphically in Figure 4.

It may be noted from Figure 4 that with an increase in asphaltene and resin contents during laboratory and field aging, the CII value also increases, making the binder stiffer and brittle and producing a sol structure as the evidenced highest value for RB in line with the literature [11]. The fractional contents of RB were restored considerably after rejuvenation through the optimum dose of Cereclor along with applying the aging process, and, again, shifted it toward the gel structure. The effect of sulfur with the aging process also contributed and it is depicted that the CII values are still less than those of VBLTA.

### 4.3. Flow Characterization of Virgin, Aged, and Rejuvenated Aged Binders

**Figure 5:**
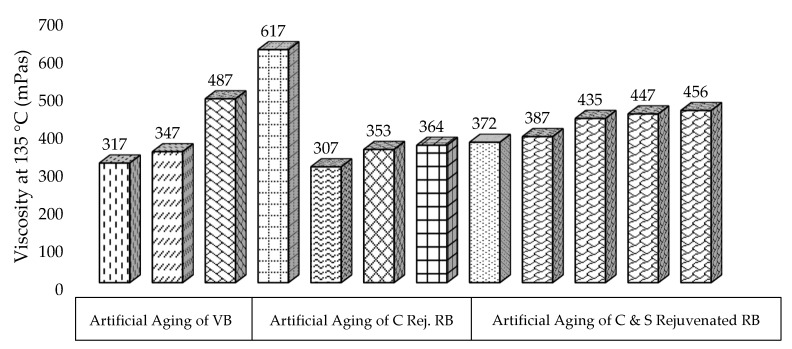
Effect of rejuvenation and aging on the viscosity of binders.

The viscosity of virgin, aged and rejuvenated aged samples was measured and is provided in Figure 5. It allows the prediction of the quality through the pumping, mixing, and laying of asphalt in the actual field construction and performance.

It may be noted from Figure 5 that the viscosity of VB is slightly higher than 300 mPas and significantly increases for VBSTA, VBLTA, and RB to depict lab and field aging. The highest value in RB is due to the stiffening of various environmental factors for degradation, making it stiffer and harder. However, through the addition of the rejuvenator and applying laboratory aging to RB, viscosity decreases significantly and still contains a lower value from VBSTA and VBLTA. A similar trend is noted and resembles physical testing and the SARA fraction of the binder. Furthermore, the addition of sulfur in the rejuvenated RB and aging also increases the viscosity due to partial decomposition of the polysulfides and crosslinking of the constituent distribution in a colloidal system according to the compositional harmonic theory of asphalt binder. Still, these values are lower than the VBLTA, which reflects the efficiency of the rejuvenation mechanism. These results also resemble the physical properties and SARA fractionations.

### 4.4. Fourier-Transform Infrared Spectroscopy (FTIR) of Virgin, Aged, and Rejuvenated Aged Binders

For this research, the FTIR (6700 Nicolet) spectrometer was used on the prepared samples. In the previous studies of FTIR spectra of asphalt binders, investigators mainly recognized two functional chemical groups produced during aging through oxidation at the intensity of peaks for carbonyl at 1700 cm^−1^ and sulfoxide at 1030 cm^−1^ [14,37,38]. The aliphatic groups are used as a reference as it is estimated that these groups are stable and do not change due to aging [39].

**Figure 6:**
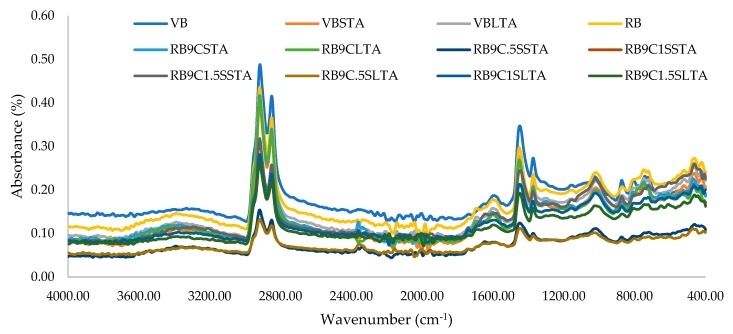
Effect of rejuvenation and aging on FTIR spectra of binders.

The FTIR absorbance versus wavenumber was plotted for the all samples, as shown in Figure 6, at different peaks.

It can be noted from Figure 6 that carbonyl (C=O) and sulfoxide (S=O) groups are changed in samples after applying the artificial aging at their associated groups. The trend of oxidation is quite different. The oxidation of methylene and degradation of unsaturated chains and/or naphthenic rings of benzene molecules promote the production of ketone and carboxylic acid groups, which ultimately increase the carbonyls. As the chain scission has started, it enhances the content of the CH_3_ group that increases the polarity of the asphalt binder. It also increases the molecular association and promotes higher asphaltene content. The height of different peaks can also vary due to the absorbance level at the relative intensity of bonds for each sample. Meanwhile, through the addition of sulfur, C=S and S=O bond intensities also increase in the spectrum in the range of 600–1250 cm^−1^ and confirms the cross-linking in the rejuvenated asphalt binder, as hypothesized in the methodology.

**Figure 7:**
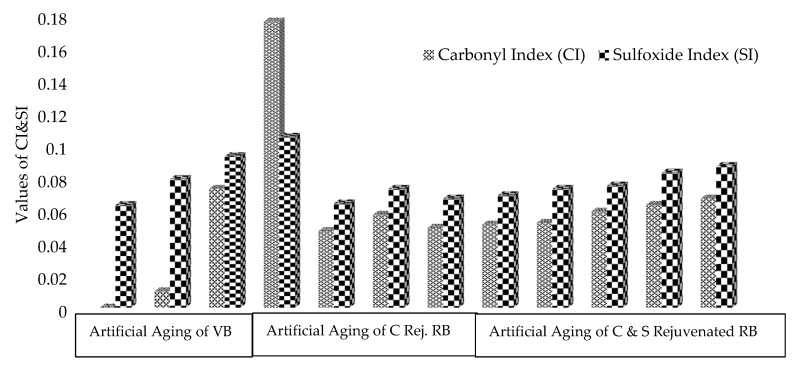
Effect of rejuvenation and aging on carbonyl and sulfoxide indices of binders.

Furthermore, for quantitative analysis, peak areas of relevant functional groups were utilized to calculate the carbonyl and sulfoxide indices, as shown in Figure 7.

It may be noted from Figure 7 that no or limited carbonyls are formed after applying artificial aging through RTFO, while sulfoxide shows an increasing trend. This inclination can be described by the fact that sulfur is more reactive than carbon in asphalt binder. The major formation of carbonyls starts during artificial aging through PAV and contains the highest value in the RB sample. As the RB is rejuvenated through an optimum dose of Cereclor, with the application of artificial aging, it has reduced both indices, but the change in carbonyls is significant. Still, these indices are lower than those of the VBLTA and RB samples. The rejuvenated samples show significant resistance against aging. This may have happened due to changes in the constituents of the dispersion or colloidal system of asphalt binder as changes have occurred through SARA fractions. The addition of sulfur in the rejuvenated binder has no significant effect on carbonyls, while a minor increase in sulfoxides also occurs due to the formation of cross-linkage bonds in C–S. This trend confirms and is in line with the literature [39].

**Figure 8:**
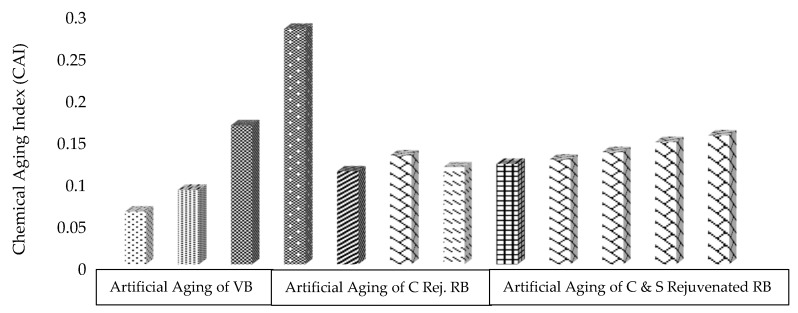
Effect of rejuvenation and aging on the chemical aging index (CAI) of binders.

The sum of both indexes (CI + SI) is utilized to calculate the chemical aging index (CAI), as shown in Figure 8. The CAI is a more specific criterion to detect the chemical susceptibility of asphalt binders against aging. The larger part of C–H bonds generally focuses on wavenumbers from 2000 to 4000 cm^−1^. Hence, aging phenomena in asphalt binders mainly concentrated on wavenumbers from 600 to 2000 cm^−1^, which is in line with the literature [14,40].

It can be noted from Figure 8 that CAI increases in VBSTA and VBLTA with a maximum value in RB due to the formation of carbonyls and sulfoxides during field aging. Furthermore, its value has been decreased significantly through the addition of rejuvenator and, by applying the aging process, still exhibits lower values than VBLTA and RB. This may have happened due to the formation of C–Cl bonds in the colloidal solution system of asphalt binder, which may rearrange the asphaltene/maltene fractions and also reduce the viscosity. Furthermore, it can be noted that the rejuvenation system has significantly resisted artificial aging.

### 4.5. Rheological Characterization of Virgin, Aged, and Rejuvenated Aged Binders

**Figure 9:**
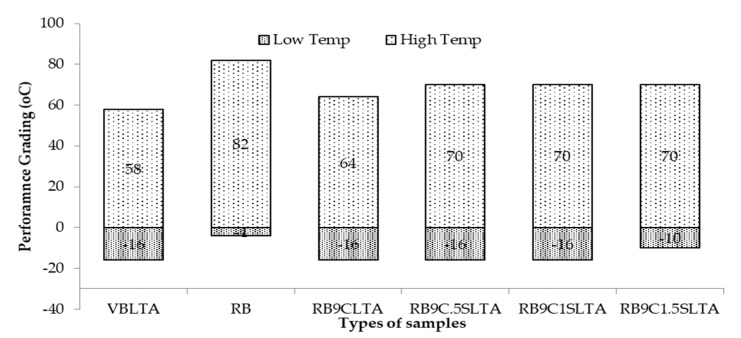
Effect of rejuvenation and aging on PG grading of asphalt binders.

Low-temperature grade: The bending beam rheometer (BBR) test was performed on prepared samples and the flexural creep stiffness was evaluated to measure the low-temperature-cracking critical temperature. RB and high-RAP-content blends, in fact, show excessive stiffness values and lower the cracking resistance especially at flexible pavements in cold areas. The critical temperature was calculated to be the lowermost temperature to exhibit flexural stiffness (S) ≤ 300 MPa, or a relaxation factor (m-value) ≥ 0.300 to measure the low-temperature grade in the PG grading, as shown in Figure 9.

It can be noted that the VBLTA sample failed at the critical temperature at 15.5 °C, and its low-temperature grade was −16 °C. RB failed too early, showing the low-temperature grade at −4 °C due to the formation of nano-agglomerates of constituents and higher fractions of asphaltene in a colloidal system of asphalt binder. Through the addition of rejuvenator and sulfur in different samples, it significantly lowered the critical temperature, and their low-temperature grades were bumped up to parent binder grades. However, a low-temperature grade of sample RB9C1.5SLTA was measured −10 °C, which is still lower than that of RB and resists low-temperature cracking more effectively than the RB. The entire scenario in increasing the low-temperature grades of rejuvenated samples has significant contribution resistance to the initiation or development of low-temperature cracking. It can happen due to restoring or rearranging the asphaltene and maltene phases in the dispersion system of asphalt binder and the ability to soften the aged binders, as witnessed in PI, SARA fraction, and aging resistance in FTIR spectral indices.

High-temperature grade: PG grading was performed on the dynamic shear rheometer (DSR) to determine the upper-temperature grade of different samples of asphalt binder in accordance with AASHTO M320. It measured the high-temperature grade in strain-controlled mode (10% strain) at a frequency level of 10 rad/s. The specimen response to sinusoidal stress was measured in the form of a complex shear modulus (G*) and a phase angle (δ). The determined binder performance grades (PGs) on different samples are shown in Figure 9.

It can be noted from Figure 9 that the PG grade of RB was ’82-4.’ Through the addition of the rejuvenator, it can be observed that the PG grades shifted three grades at the higher and two grades at the lower temperature, i.e., from ’82-4’ to ’64-16,’ confirming the significant rejuvenator effect on the rejuvenation of aged binder. This observation also proposes that if only the performance grade is considered, the dosage amount of the rejuvenator can be varied to measure the rejuvenating effect at both upper and lower temperatures to meet the selected binder specification. However, with the addition of different doses of sulfur, upper PG grades shifted through one grade from ’64 to 70,’ which is still softer than RB because it is two grades lower than RB. The effectiveness of the rejuvenator also depends on aging conditions and, consequently, the distribution of constituents in the colloidal system of aged binders.

**Figure 10:**
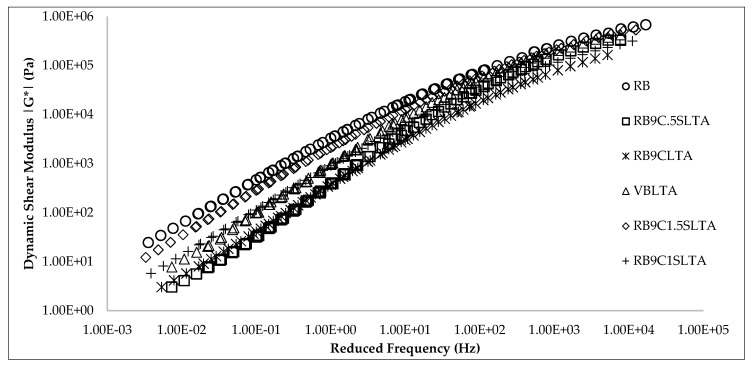
Effect of rejuvenation and aging on the master curve of binders.

Frequency sweep test was conducted on all samples and, according to the working principle, it applies oscillatory shear stress at a constant strain level, and the storage and loss modulus are taken at allowable temperatures and frequency ranges. Furthermore, this data was fitted to construct master curves using sigmoidal functions, as designated by the mechanistic empirical pavement design guide (MEPDG). Master curves were constructed using two replicates of each blend of material at the reference temperature of 58 °C by providing a horizontal shift in the data attained from each test temperature. The master curves of binders at 58 °C for different samples after applying artificial aging are shown in Figure 10.

It may be noted from the master curves in Figure 10 that RB has the highest stiffness at low and high frequencies than all the other samples due to the oxidation process that promotes higher aging. This trend is also observed with the SARA fractions, as the aging period increases from laboratory to field aging; the asphalt binder becomes stiffer and viscous due to the change in the entire constituents of asphalt binder. This leads to the enhancement of the asphaltene contents that propagate the agglomeration of the molecular structure and reduction in maltene contents in an overall colloidal system of a binder. Furthermore, it accelerates the oxidative aging of asphalt binders. However, this effect drops at lower temperature. Through the addition of rejuvenator and applying the aging process, it reduces the stiffness of RB and improves its resistance against cracking, as well as its viscous properties in different trends. At low frequency, a significant reduction in stiffness is observed that reflects the moderate to high temperature regions. In actuality, the parent grade of RB binder is mostly utilized in these regions, so this rejuvenator can be used to rejuvenate it significantly. With the addition of different dosages of sulfur in rejuvenated RB, the stiffness increased with the |G*| value of these samples but was still less than that of RB. It shows that the addition of sulfur improved the stiffness of the samples at higher temperatures and frequency, which indicates the improvement in the binder’s higher-temperature properties and resistance against the non-structural rutting due to heavy loading and high-temperature ranges.

### 4.6. Correlations between Physical, Rheological, and Chemical Parameters of Asphalt Binder

After conducting and explaining the results of physical, chemical, and rheological testing on different samples of asphalt binder, an additional analysis was carried out at IBM SPSS Statistics 22 to detect any suitable correlation between these parameters. In this analysis, different parameters were selected such as penetration, ductility, softening point, penetration index, and viscosity from the physical and asphaltene content, the colloidal index from SARA fractions, and the PG upper grade from rheological properties. Finally, chemical parameters such as carbonyl, sulfoxide, and chemical aging indexes were taken from the FTIR bands of different samples. Initially, parameters were tabulated, and descriptive statistics was applied to calculate the mean value and standard deviation on each sample. Then, the correlation matrix was applied, as shown in Table 3.

Table 3 demonstrates the linear correlation coefficients (r) between an individual pair of variables assessed. This coefficient is a dimensionless parameter that can fluctuate between −1.0 and 1.0 and designates the scale of the expected correlation between each pair of the data. It indicates the positive trend when a pair of data increase at the same time, whereas a negative value represents possible opposite trends. The null correlation depicts that the pair of data are not linearly dependent on each other. It illustrates the various medium and high significance type correlations between physical, rheological, and chemical parameters for different samples of asphalt binders. It can be noted that most parameters are directly related as one increase, the other also increases. However, a few parameters are inversely correlated.

**Figure 11:**
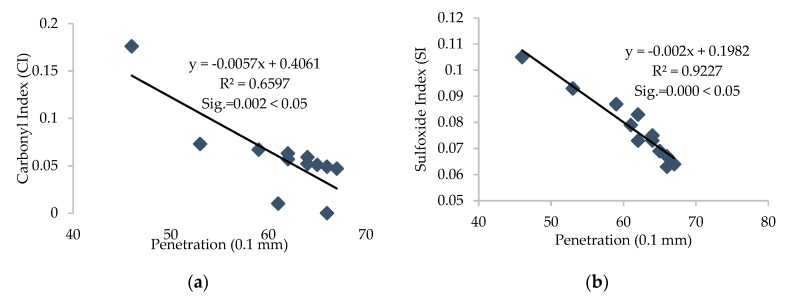
(**a**) Correlation between FTIR indices and (**b**) penetration values of all samples of binders.

**Figure 12:**
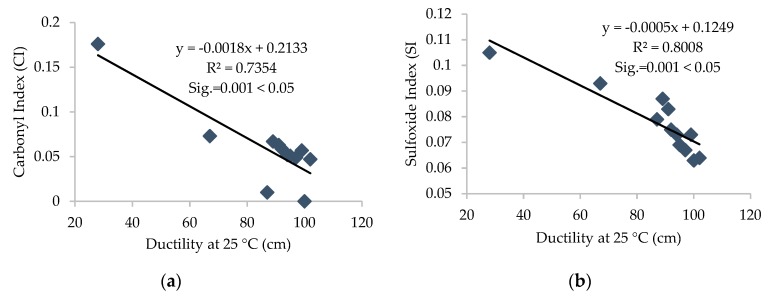
(**a**) Correlation between FTIR indices and (**b**) ductility values of all samples of binders.

**Figure 13:**
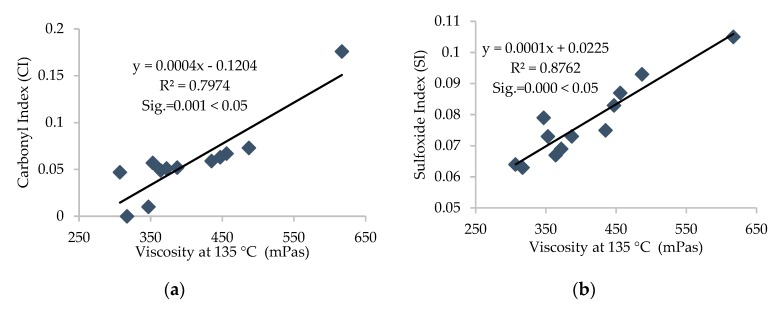
(**a**) Correlation between FTIR indices and (**b**) viscosity of all samples of binders.

**Figure 14:**
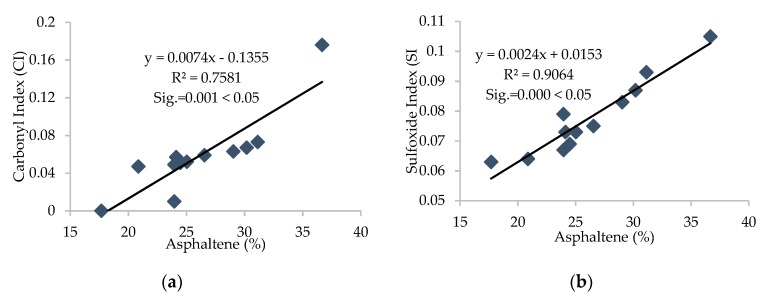
(**a**) Correlation between FTIR indices and (**b**) asphaltene content of all samples of binders.

**Figure 15:**
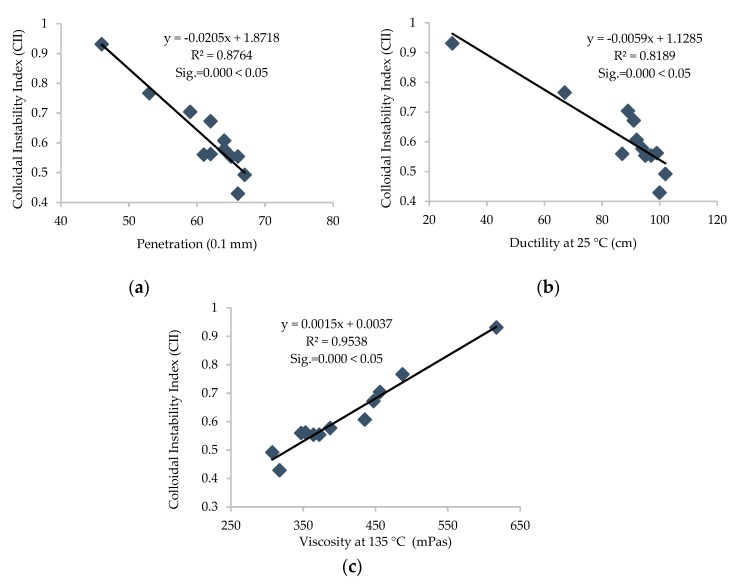
Correlation between CII and physical properties of all samples of binders. (**a**) Penetration (0.1 mm), (**b**) Ductility at 25 °C (cm), (**c**) Viscosity at 135 °C (mPas).

Furthermore, the R^2^ value was calculated for each correlation between different parameters, and linear correlations with relatively high R^2^ were established and are presented in Figure 11, Figure 12, Figure 13, Figure 14 and Figure 15.

It can be depicted how the difference in chemical parameters can lower or increase the physical-rheological parameters. In addition, analysis of variance (ANOVA) was conducted for different regression equations, and significant values (Sig.) for every linear regression model were established and are presented in Figure 12, Figure 13, Figure 14 and Figure 15. It can be noted that the (Sig.) values are less than 0.05 for all pairs of different parameters, so the correlations are statistically valid [17].

It can be noted from Figure 11, Figure 12 and Figure 13 that carbonyl and sulfoxide indices showed a decreasing trend with penetration and ductility and moderate-to-high correlation, respectively. These indices showed an increasing trend and high correlation with viscosity for all samples of asphalt binder. Moreover, Figure 14 depicts that the FTIR indices increased with higher asphaltene content in the SARA fractions and indicates high correlation. Furthermore, it can be noted from Figure 15 that penetration and ductility showed decreasing, and viscosity showed increasing, trends with the increase in colloidal instability index and high correlation. It indicates that the aging resistance of asphalt binder increases with the addition of rejuvenator after applying the artificial aging, which was also seen with the SARA fraction and FTIR results.

## 5. Conclusions

In this study, different test methods were conducted to investigate the effects of a new rejuvenator on physical, SARA fractionation, rheological, and chemical changes on rejuvenated blends of RAP binder after applying artificial aging. From the test results, the following conclusions can be drawn:

1. The SARA test results showed significant changes in the colloidal structure of aged and rejuvenated aged binder mixes. In the virgin binder, artificial aging increased the asphaltene contents and reduced the aromatics. However, this trend was more significant in the RAP binder due to the drastic effect of aging. The rejuvenator substantially restored its colloidal structure due to the change in asphaltene and maltene contents, as well as varying the locations of constituents and the more stable structure, even after applying artificial aging.
2. During the aging of asphalt binder, the oxidation of methylene and degradation of unsaturated chains and naphthenic rings of benzene molecules promoted the production of ketone and carboxylic acid groups, which ultimately increased the carbonyls and sulfoxide that contribute to higher stiffness and low-temperature cracking. The addition of Cereclor reduced the carbonyls and sulfoxides of the aged binder, enhanced its resistance to aging, and would contribute to the long-term performance.
3. From DSR test results, it has been observed that the rejuvenator shifted PG grades in both the upper and lower temperatures, i.e., from ’82-4’ to ’64-16,’ confirming the significant rejuvenation effect on the aged binder. Furthermore, increasing the low-temperature grades had a significant effect on the resistance to initiation or development of low-temperature cracking and permanent deformation at higher temperatures. This observation also suggests that if only performance grade is considered, the dosage amount of the rejuvenator can be varied to measure the rejuvenating effect at a wide range of temperatures to meet the binder specification.
4. It can be concluded that relatively good correlations exist between physical, chemical, and rheological properties of asphalt binder, and more accurate correlations can be established with more data and a list of experiments. Such correlations may be useful to explore the quick and unknown parameters of asphalt binders.

## Figures and Tables

**Table 1:** Physical properties of a virgin and aged binder, and Cereclor and sulfur.

Property	Virgin Binder	Aged Binder	Cereclor	Sulfur
Penetration (0.1 mm)	66	46	Chlorine Content (%): 70	−
Softening Point (°C)	51	67	Viscosity at 100 °C (Cp): 86.2	Melting Point (°C): 115.21
Ductility at 25 °C (cm)	100	28	Pour Index Approx. (°C): + 20	Boiling Point (°C): 444.61
Viscosity at 135 °C (mPas)	317	617	Stability after 4 h 175 °C: 0.2	Density (g cm^−3^): 2.07

**Table 2:** Conventional properties of different asphalt binder samples.

Sr. No	Binder Matrix	Basic Physical Tests on Asphalt Binder			
		Penetration Test at 25 °C (AASHTO T49) (dmm)	Softening Point (AASHTO T 53) (°C)	Ductility Test (AASHTO T51) (cm)	Specific Gravity Test @ 25 °C (g/cm^3^)
1	VB	66	51	100	1.01
2	VBSTA	61	55	87	1.02
3	VBLTA	51	61	67	1.03
4	RB	46	67	28	1.06
−	RB9C (without aging)	71	50	112	1.00
5	RB9CSTA	68	53	102	0.99
6	RB9CLTA	63	55	99	0.98
7	RB9C0.5SSTA	66	54	97	1.01
8	RB9C1SSTA	65	55	95	1.012
9	RB9C1.5SSTA	64	56	94	1.014
10	RB9C0.5SLTA	64	57	92	1.013
11	RB9C1SLTA	62	58	91	1.015
12	RB9C1.5SLTA	59	58	89	1.02

**Table 3:** Correlation matrix of different parameters of asphalt binders.

CORRELATION	Pen.	SP	Duct.	PI	AC	CII	Vis.	CI	SI	CAI	PG-Upper
Pen.	1.00	−0.87	0.96	−0.84	−0.88	−0.94	−0.89	−0.81	−0.96	−0.88	−0.71
sp	−0.87	1.00	−0.86	0.96	0.97	0.97	0.97	0.86	0.92	0.91	0.83
Duct.	0.95	−0.86	1.00	−0.83	−0.83	−0.91	−0.89	0.85	−0.89	−0.90	−0.74
pi	−0.83	0.96	−0.83	1.00	0.97	0.96	0.95	0.90	0.90	0.93	0.89
ac	−0.88	0.97	−0.83	0.97	1.00	0.98	0.96	0.87	0.95	0.92	0.87
cii	−0.93	0.97	−0.90	0.96	0.98	1.00	0.97	0.89	0.97	0.94	0.85
Vis.	−0.89	0.97	−0.89	0.95	0.96	0.97	1.00	0.89	0.93	0.93	0.91
ci	−0.81	0.87	−0.85	0.90	0.87	0.89	0.89	1.00	0.79	0.99	0.90
si	−0.96	0.93	−0.89	0.90	0.95	0.97	0.93	0.79	1.00	0.87	0.77
cai	−0.88	0.91	−0.90	0.93	0.92	0.94	0.93	0.99	0.87	1.00	0.91
pg-Upper	−0.71	0.84	−0.74	0.89	0.87	0.85	0.91	0.90	0.77	0.91	1.00
